# Long-term survival in a child with severe encephalopathy, multiple respiratory chain deficiency and *GFM1* mutations

**DOI:** 10.3389/fgene.2015.00102

**Published:** 2015-03-23

**Authors:** Sara Brito, Kyle Thompson, Jaume Campistol, Jaime Colomer, Steven A. Hardy, Langping He, Ana Fernández-Marmiesse, Lourdes Palacios, Cristina Jou, Cecilia Jiménez-Mallebrera, Judith Armstrong, Raquel Montero, Rafael Artuch, Christin Tischner, Tina Wenz, Robert McFarland, Robert W. Taylor

**Affiliations:** ^1^Serviço de Pediatria, Centro Hospitalar de Leiria, Hospital de Santo AndréLeiria, Portugal; ^2^Neuromuscular Unit, Neuropaediatrics Department, Hospital Sant Joan de DéuBarcelona, Spain; ^3^Wellcome Trust Centre for Mitochondrial Research, Institute of Neuroscience, Newcastle UniversityNewcastle upon Tyne, UK; ^4^Centro de Investigación Biomédica en Red de Enfermedades Raras, Instituto de Salud Carlos IIIBarcelona, Spain; ^5^Diagnosis and Treatment Unit for Inborn Errors of Metabolism, Hospital Clínico Universitario de Santiago de CompostelaLa Coruña, Spain; ^6^Progenika Biopharma a Grifols CompanyDerio, Spain; ^7^Pathology Department, Hospital Sant Joan de DéuEsplugues Barcelona, Spain; ^8^Biochemical, Genetics and Rett Unit, Laboratory Department, Hospital Sant Joan de DéuEsplugues Barcelona, Spain; ^9^Biochemical Department, Hospital Sant Joan de DéuEsplugues Barcelona, Spain; ^10^Cluster of Excellence: Cellular Stress Responses in Aging-Associated Diseases (CECAD), Institute for Genetics, University of CologneCologne, Germany; ^11^German Network for Mitochondrial Disorders (mitoNET)Munich, Germany

**Keywords:** *GFM1*, mtEFG1, mitochondrial disorders, brain MRI, encephalopathy

## Abstract

**Background:** Mitochondrial diseases due to deficiencies in the mitochondrial oxidative phosphorylation system (OXPHOS) can be associated with nuclear genes involved in mitochondrial translation, causing heterogeneous early onset and often fatal phenotypes.

**Case report:** The authors describe the clinical features and diagnostic workup of an infant who presented with an early onset severe encephalopathy, spastic-dystonic tetraparesis, failure to thrive, seizures and persistent lactic acidemia. Brain imaging revealed thinning of the corpus callosum and diffuse alteration of white matter signal. Genetic investigation confirmed two novel mutations in the *GFM1* gene, encoding the mitochondrial translation elongation factor G1 (mtEFG1), resulting in combined deficiencies of OXPHOS.

**Discussion:** The patient shares multiple clinical, laboratory and radiological similarities with the 11 reported patients with mutations involving this gene, but presents with a stable clinical course without metabolic decompensations, rather than a rapidly progressive fatal course. Defects in *GFM1* gene confer high susceptibility to neurologic or hepatic dysfunction and this is, to the best of our knowledge, the first described patient who has survived beyond early childhood. Reporting of such cases is essential so as to delineate the key clinical and neuroradiological features of this disease and provide a more comprehensive view of its prognosis.

## Introduction

Mitochondrial diseases due to deficiencies in the mitochondrial oxidative phosphorylation system (OXPHOS) have an estimated birth prevalence of 1 in 10,000 (Schaefer et al., 2008). Mitochondria are semi-autonomous organelles. They contain their own genome (mtDNA), but rely on the nuclear DNA for full functionality. The nuclear genome encodes approximately 1500 mitochondrial proteins (Elstner et al., 2008) and provides essential factors for mtDNA replication, transcription, translation, and assembly of the five OXPHOS complexes. Human mtDNA is a minimal genome and encodes only 13 essential subunits of the OXPHOS system as well as 2 rRNAs and 22 tRNAs (Anderson et al., 1981). Mitochondria possess their own translation system to synthesize the mtDNA-encoded proteins. With the exception of complex II, all OXPHOS enzymes derive from dual genetic origin with at least one subunit encoded by the mtDNA, thus, mitochondrial protein synthesis is crucial for OXPHOS function and ATP generation. It is therefore not surprising, that defects in the mitochondrial translation machinery are an emerging and increasingly recognized group of mitochondrial disorders (Jacobs and Turnbull, 2005; Scaglia and Wong, 2008; Smits et al., 2010; Kemp et al., 2011). Defects in mitochondrial protein translation are hypothesized to be the primary cause of combined OXPHOS deficiencies, in which the activity of more than one OXPHOS complex is compromised (Smits et al., 2010; Kemp et al., 2011; Rotig, 2011). New sequencing technologies have rapidly increased the canon of genes known to cause combined respiratory chain deficiency, an effect largely exerted through disrupted mitochondrial DNA maintenance, nucleotide transport or various aspects of translation including tRNA modification and aminoacylation (Taylor et al., 2014).

The early genetic investigation involving patients with defective mitochondrial translation led to the description of a number of nuclear-encoded defects in initiation, elongation, and termination factors required for mitochondrial translation (Ylikallio and Suomalainen, 2012). These seem to be responsible for heterogeneous phenotypes, manifesting predominantly in tissues with high energy requirements and with early onset and rapidly progressive fatal courses (Antonicka et al., 2006; Balasubramaniam et al., 2012).

Here we describe the clinical features and diagnostic workup of an infant who presented with an early onset severe encephalopathy resulting from combined deficiencies of OXPHOS due to recessive mutations (including one novel variant) in a nuclear gene (*GFM1*), encoding mitochondrial translation elongation factor G1 (mtEFG1) and compare these findings with other reported cases.

## Background

### Case report

**Figure 1:**
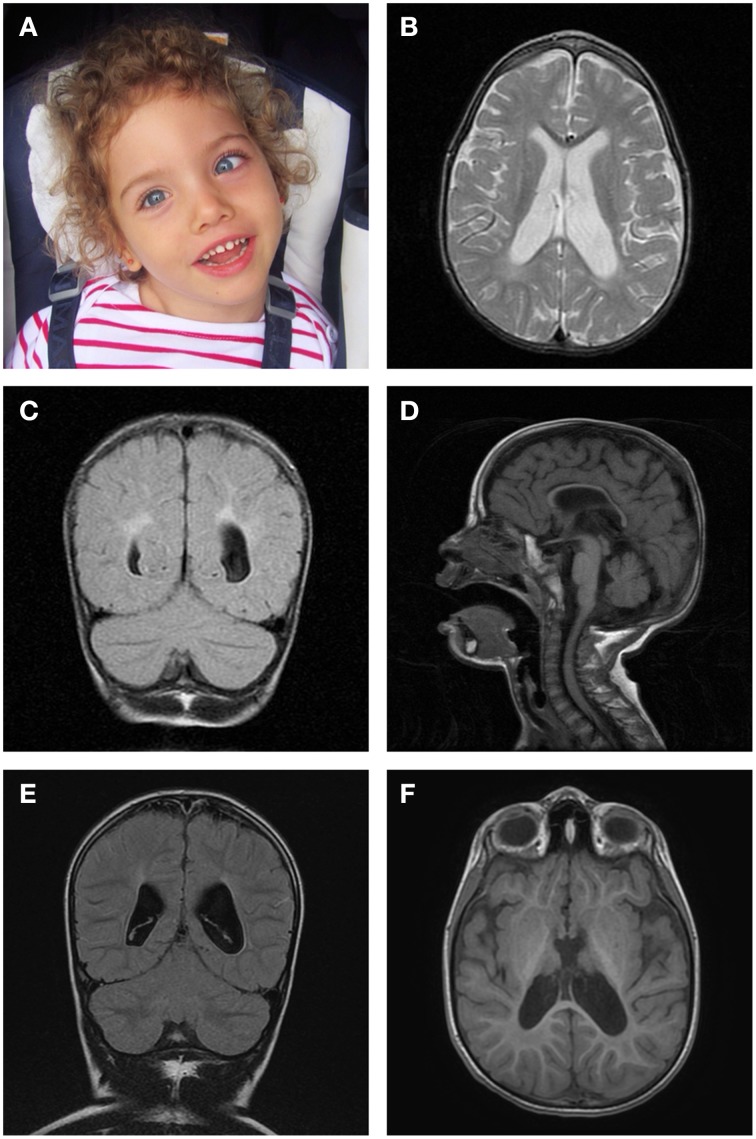
**Brain magnetic resonance imaging. (A)** Photograph of the patient aged 5 years demonstrating left convergent squint and normal facial appearance. **(B)** Axial T2-weighted image at 29 months, revealing enlarged lateral ventricles. **(C)** Coronal T2-FLAIR image at 29 months, displaying abnormal white matter signal in a periventricular distribution. **(D)** Midsagittal T1-FLAIR image at 29 months, showing thinning of the corpus callosum with enlarged subarachnoid spaces. **(E)** Coronal T2-FLAIR image at 5 years 6 months, showing abnormal periatrial signal and enlarged lateral ventricles. **(F)** Axial 3D FSPGR image at 5 years 6 months, displaying abnormal signal in the posterolateral region of the putamen nucleus.

This female patient (Figure 1A), currently 5 years 6 months old, was born to unrelated Caucasian parents following a pregnancy that was uneventful until 36 weeks gestation, when acute oligohydramnios and late presenting breech were diagnosed. Cesarean section was performed without complication and no neonatal resuscitation was required. Birth weight was on the 50th centile, length on the 10th and head circumference on the 97th centile. Neonatal metabolic and hearing screenings were normal. At 2 months old, she developed paroxysms of irritability that evolved into a persistently irritable state with inconsolable crying, opisthotonic posturing, feeding difficulties, and failure to thrive. At presentation, she had severe axial hypotonia with poor head control, spasticity of extremities and severely impaired visual tracking; there were no noticeable dysmorphic features. At 7 months old, she was already developmentally delayed prior to the onset of infantile spasms, which showed prompt clinical resolution with medication, although electrical abnormalities persisted on the electroencephalogram. She gained eye contact and social smile, but has never been able to sit independently and has not acquired verbal language skills. She has developed a spastic-dystonic tetraparesis with dystonic movements affecting her face, particularly her mouth.

After excluding perinatal metabolic and infectious causes, further investigation confirmed persistently elevated lactate levels in blood (maximum 4.87 mmol/L; normal range 0.77–2.44 mmol/L) and cerebral spinal fluid (CSF; 4.79 mmol/L; normal range 1.11–2.22 mmol/L), along with elevated blood pyruvate (maximum 0.15 mmol/L; normal range 0.03–0.10 mmol/L) and alanine (maximum 742 umol/L; normal range 226–416 umol/L). There was no hepatic dysfunction. The remaining blood, CSF and urine analysis were unremarkable.

Brain magnetic resonance revealed widened subarachnoid spaces, Sylvian sulcus and ventricular system; diffuse and non-specific alteration of white matter signal and thinning of the corpus callosum (Figures 1B–D). These findings were only slowly progressive and apparently unchanged on repeat cranial MR imaging at 16 and 29 months old. Spectroscopy showed no significant changes for age, including lactate. However, repeat MRI performed at 5 years 6 months old did show some further ventricular enlargement (Figure 1E) and abnormal signal change in the posterolateral putamen (Figure 1F).

Electroencephalography showed a poorly formed background with multifocal polyspike paroxysms. Visual evoked potentials revealed delayed conduction and brainstem auditory evoked potentials indicated bilateral vestibulocochlear nerve involvement. Electromyography demonstrated discrete involvement of the lower limb motor axons without demyelination. Muscle and skin biopsies were performed to investigate a likely mitochondrial disorder.

### Materials and methods

All procedures followed were in accordance with the ethical standards of the responsible committee on human experimentation (institutional and national) and with the Helsinki Declaration of 1975, as revised in 2000. Informed consent was obtained from the parents of the patient, including consent about the publication of identifying information.

#### Histopathological and biochemical studies

A diagnostic muscle biopsy (quadriceps) was orientated, frozen and processed for histology and histochemistry following standard procedures. Respiratory chain complex activities were measured and normalized to citrate synthase activity in cultured fibroblasts as previously described (Kirby et al., 2007).

#### Genetic studies

##### Mitochondrial DNA analysis

Real-time PCR analysis of mtDNA levels was undertaken in muscle DNA to investigate a possible mtDNA depletion syndrome. Southern blot analysis of mtDNA linearized following *Pvu*II digestion was performed using a probe obtained by amplification of a region of the human *MT-RNR1* gene, whilst mtDNA rearrangements were also screened by long-range PCR. The sequence of the entire mitochondrial genome was determined in muscle using standard protocols.

##### Next generation sequencing of nuclear-mitochondrial genes

Simultaneous sequencing of the coding regions (exons and exon-intron junctions) of 150 nuclear-encoded genes associated with mitochondrial respiratory chain defects was performed using next generation sequencing technology consisting of an in-solution hybridization enrichment method with a custom Sure Select XT kit (Agilent Technologies) and subsequent sequencing in MiSeq platform (Illumina).

The custom Sure Select oligonucleotide probe library was designed including all transcripts from each target gene. Sequence capture, enrichment, and elution were performed according to the manufacturer's instructions. Captured fragments were sequenced in pair-end 100-base mode using Miseq (Illumina) platform. Image analysis and processing of the fluorescence intensities in sequences (“Base Calling”) was performed with Real Time Analysis (RTA) software 1.8.70 (Illumina), and quality control of the data was developed with FastQC v0.10.1 program (http://www.bioinformatics.bbsrc.ac.uk/projects/fastqc/). Reads were aligned to the reference genome GRCh37 with BWA v0.7.5a software (Li and Durbin, 2009). NGSrich v0.7.5 software (http://ngsrich.sourceforge.net) used as a control previous to variant detection, and BEDTools 2.17.0 (http://bedtools.readthedocs.org/en/latest/#) and Picard 1.93 (http://picard.sourceforge.net) for intermediate steps. Varscan and SAMtools v0.1.19 (Li et al., 2009) were the variant detection software used and Annovar Nov2011 (Wang et al., 2010) for variant annotation. The assay reached a mean coverage for the *GFM1* gene of 240X and 313X for exons 12 and 16 respectively.

Sanger sequencing was used to confirm the mutations identified in the proband and determine phase. Exons 12 and 16 of *GFM1* gene (NM_024996) were amplified with specific primers designed using the free software Primer3 v.0.4.0 (www.frodo.wi.mit.edu/) and the amplified fragments sequenced using standard methodologies. Sequencing reactions consisted of 1.0 μl of previously purified PCR products (ExoSAP-IT, USB, Cleveland, OH), 1 μl of each primer, and 1 μl of Big Dye Terminator v3.1 from the Cycle Sequencing kit (Applied Biosystems, Foster City, CA). The reactions were run on an ABI 3730 DNA Analyzer (Applied Biosystems, Foster City, CA). Analysis was performed with the Staden package free software.

#### Cell preparation and western blot analysis

Human fibroblasts were trypsinized, pelleted and resuspended in cell lysis buffer [50 mM Tris-HCl pH 7.5, 130 mM NaCl, 2 mM MgCl_2_, 1% Nonidet P-40, 1 mM PMSF and protease inhibitor cocktail (Roche)]. Cell lysates were vortexed briefly, centrifuged at 500 *g* for 5 min at 4°C and the supernatant retained.

Mitochondrial fractions were prepared from cell pellets resuspended in homogenization buffer (0.6M mannitol, 10 mM Tris-HCl pH7.4, 1 mM EGTA, 0.1% BSA, and 1 mM PMSF) and hand homogenized by 15 passes in a Teflon:glass dounce homogenizer. Following centrifugation at 400 *g* the supernatant was retained and the pellet was re-homogenized and centrifuged as before. The combined supernatants were cleared at 400 *g* and then the crude mitochondria pelleted at 11,000 *g*.

Cell lysates and mitochondrial lysates were incubated with sample dissociation buffer (final concentrations: 6.25 mM Tris/HCl pH 6.8, 2% SDS, 10% glycerol 0.01% bromophenol blue and100 mM DTT) for 30 min at 37°C, separated by 12% SDS–PAGE and immobilized by wet transfer (100 V, 1 h at 4°C) on to PVDF membrane (Immobilon-P, Millipore Corporation) in 25 mM Tris, 192 mM glycine, 0.02% SDS, and 15% methanol. Proteins of interest were bound by overnight incubation at 4°C with antibodies against COXI (Abcam ab14705), SDHA (Abcam ab14715), Porin/VDAC1 (Abcam ab14734), mtEFG1 (Abcam ab173529), and NDUFB8 (Abcam ab110242) followed by HRP-conjugated secondary antibodies (Dako Cytomation) and visualized using ECL-prime (GE Healthcare) and BioRad ChemiDoc MP with Image Lab software.

#### De novo mitochondrial protein synthesis

Mitochondrial protein synthesis in cultured cells was performed essentially as described previously (Chomyn, 1996). Fibroblasts were labeled for 1 h in methionine/cysteine free DMEM (Sigma) with 200 uCi/ml of a [^35^S]-methionine/cysteine mixture (Perkin Elmer) and 100 ug/ml emetine dihydrochloride (Sigma) followed by 10 min chase in standard DMEM with additional 7.5 ug/ml cold methionine. Aliquots (50 ug) of total cell protein were separated by 15% SDS–PAGE. Signals were detected using the Typhoon FLA9500 Phosphorimager and ImageQuant software (GE Healthcare).

### Genetic, pathological, and biochemical results

Genetic studies were performed sequentially in accordance with clinical suspicion and showed a 46, XX normal karyotype, fluorescent *in situ* hybridization (FISH) excluded Wolf-Hirschhorn syndrome, direct sequencing of the *FOXG1* gene was normal, multiplex ligation-dependent probe amplification (MLPA) for subtelomeric deletions and for *CDKL5*, *NTNG1*, and *ARX* genes was unremarkable. The activity of pyruvate dehydrogenase was normal in fibroblasts.

**Figure 2:**
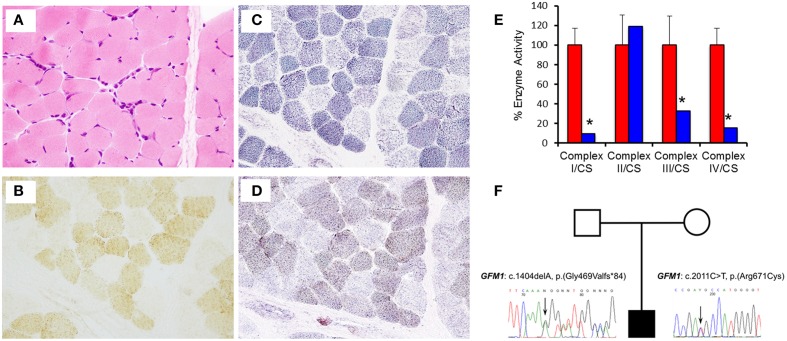
**Muscle histology and histochemistry reveals mitochondrial abnormalities. (A)** Haematoxylin and Eosin (H&E) histology shows mild variation in fiber size. **(B)** Cytochrome *c* oxidase (COX) histocytochemical activity revealed a mosaic pattern of COX deficiency without subsarcolemmal mitochondrial aggregates. **(C)** Succinate dehydrogenase (SDH) reaction indicates a population of pale fibers within the section. **(D)** Sequential COX-SDH histochemistry shows a population of weak “blue” fibers corresponding to the COX-deficient, SDH-positive fibers detected following the individual enzyme reactions. **(E)** The assessment of individual respiratory chain enzyme activities in fibroblasts identified a severe OXPHOS deficiency affecting complex I, III, and IV in the patient (blue) compared with controls (red). Mean enzyme activities of control fibroblasts (*n* = 8) are set at 100% and error bars represent the standard deviation. ^*^denotes values outside of the normal range. **(F)** Pedigree and sequence analysis of the two mutations identified in the patient.

Muscle histology and histochemistry showed no evidence of obvious subsarcolemmal mitochondrial accumulation (ragged-red fibers) or inclusions and mild variation in fiber size (Figure 2A). There was a generalized decrease in histochemical cytochrome *c* oxidase (COX) activity including a population of fibers (mosaic) that were COX-deficient (Figure 2B). The succinate dehydrogenase (SDH) reaction showed a population of pale fibers (Figure 2C), whereas the sequential COX/SDH reaction highlighted a population of weakly-reacting blue (COX-deficient) fibers (Figure 2D). In addition, the size of intracellular lipid droplets stained with Sudan Black appeared to be increased (not shown). Biochemical assessment of respiratory chain enzymatic function in fibroblasts confirmed impaired complex I (0.019 units; controls (mean ± SD) 0.197 ± 0.034), complex III (0.211 units; controls (mean ± SD) 0.646 ± 0.192), and complex IV (0.165 units; controls (mean ± SD) 1.083 ± 0.186) activities, with a low complex I:II ratio (0.071; range 0.58–0.90) consistent with a generalized defect of mitochondrial translation (Figure 2E). The activity of complex V was not assessed.

Mitochondrial DNA copy number abnormalities and large-scale rearrangements were excluded in muscle; complete sequencing of the mitochondrial genome in this tissue identified several well-characterized polymorphic variants, but no plausible pathogenic mtDNA mutation.

Skeletal muscle DNA was used to perform genetic screening for mitochondrial encephalopathy, with simultaneous next generation sequencing of the coding regions of 150 genes associated with mitochondrial respiratory chain disorders. This revealed two inherited, likely pathogenic heterozygous mutations: c.1404delA, p.(Gly469Valfs^*^84), in exon 12 (NM_024996.5, OMIM 606639) and c.2011C>T, p.(Arg671Cys), in exon 16 (NM_024996.5, OMIM 606639) of the *GFM1* gene (Figure 2F). The former predicts a novel frameshift mutation, whilst the latter is a reported missense mutation predicting the substitution p.(Arg671Cys) of a conserved amino acid (Calvo et al., 2012; Galmiche et al., 2012). Further analysis verified that the mother was a heterozygous carrier of the c.1404delA p.(Gly469Valfs^*^84) variant whereas the father was a heterozygous carrier of the c.2011C>T p.(Arg671Cys) variant.

**Figure 3:**
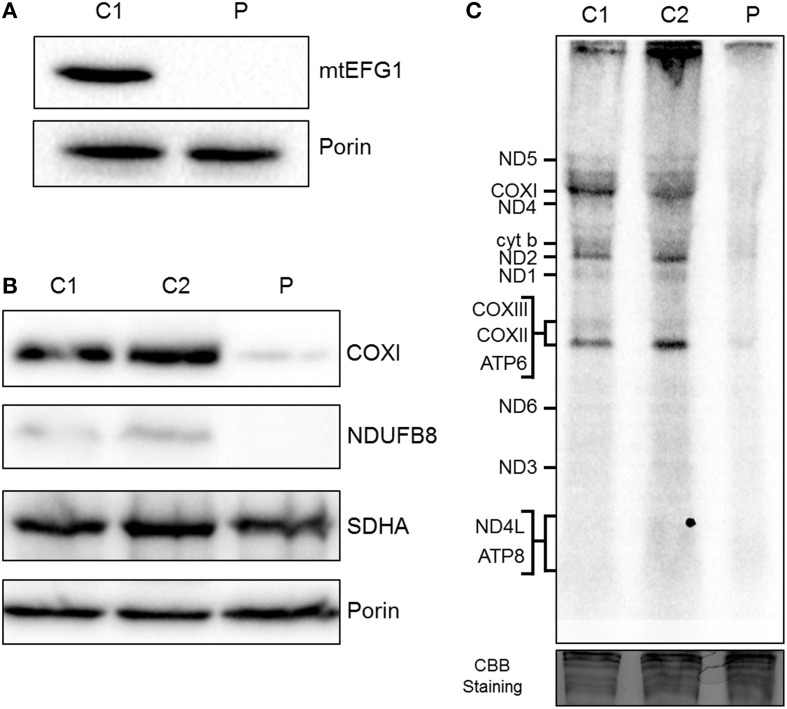
**Biochemical assessment of patient fibroblasts. (A)** Mitochondria isolated from control (C1) and patient (P) fibroblasts were subjected to SDS-PAGE and western blot analysis using an anti-mtEFG1 antibody. Anti-porin (VDAC1) antibody was used as a loading control. **(B)** Whole cell lysate from control (C1 and C2) and patient (P) primary fibroblasts were subjected to western blot analysis. Antibodies against COXI (mitochondrial encoded subunit of Complex IV), NDUFB8 (nuclear encoded subunit of Complex I), SDHA (nuclear encoded subunit of Complex II) and porin/VDAC1 (mitochondrial loading control). **(C)** Control (C1 and C2) and patient (P) fibroblasts were treated with emetine dihydrochloride to inhibit cytosolic translation and mitochondrial protein synthesis analyzed by [^35^S] met/cys incorporation (1 h followed by a 10 min chase). Cell lysate (50 μg) was separated through a 15% polyacrylamide gel. The gel was stained with Coomassie blue (CBB) to confirm equal loading. Post fixation and drying the signal was visualized by Typhoon FLA9500 PhosphorImaging. Signals were ascribed following established migration patterns (Chomyn, 1996).

Western blot analysis showed that steady state levels of mtEFG1 protein were severely decreased in isolated mitochondria from patient fibroblasts (Figure 3A) compared to control fibroblasts, confirming pathogenicity of the *GFM1* mutations. Western blot analysis of patient fibroblasts showed a reduction in the steady-state levels of NDUFB8 (complex I subunit) and COXI (complex IV subunit), but similar levels of SHDA (complex II) compared to controls (Figure 3B), which corresponds well with the complex activity data (Figure 2E). A generalized impairment of *de novo* mitochondrial protein synthesis was also observed in patient fibroblasts (Figure 3C), consistent with a decrease in translation elongation due to the reduction of mtEFG1 protein levels.

## Discussion

The nuclear gene, *GFM1*, is located at 3q25.1–q26.2 and encodes the mitochondrial translation factor mtEFG1 (Gao et al., 2001), which acts as a five domain GTPase (Valente et al., 2007; Balasubramaniam et al., 2012). It catalyzes the translocation of peptidyl-tRNA from the ribosomal acceptor aminoacyl site to the peptidyl site following peptide bond formation, with the concomitant removal of the deacylated tRNA, advancement of the mRNA by one codon and exposure of the next codon (Smits et al., 2010).

### Review of similar cases

Following the first report of recessive mutations in this gene causing early-onset mitochondrial disease and a generalized disorder of mitochondrial translation (Coenen et al., 2004), 11 clinical cases have been published in the literature (Coenen et al., 2004; Antonicka et al., 2006; Valente et al., 2007; Smits et al., 2011; Balasubramaniam et al., 2012; Calvo et al., 2012; Galmiche et al., 2012). A review of the clinical, laboratory and radiological characteristics of the reported cases is shown in Table 1, highlighting a number of similarities between our patient and those previously characterized, although there are also some notable differences.

**Table 1:** **Comparative study of clinical, laboratory and radiological findings of the patients reported with *GFM1* mutations**.

**Clinical Features**	**Reported cases**												
		**A^a^**	**B^a^**	**C^b^**	**D^b^**	**E^c^**	**F^d^**	**G^e^**	**H^f^**	**I^f^**	**J^g^**	**K^g^**	**L^h^**
Consanguinity		+	+	−	−	−	+	−	+	+	−	−	−
IUGR		+	+	+	+	−	+	+	+	−	na	na	−
Microcephaly		+	−	+	+	+	+	+	+	+	na	na	−
Dysmorphism		−	−	+	−	+	−	+	+	+	na	na	−
Feeding difficulties		−	−	−	na	+	+	−	+	−	na	na	+
Seizures		−	−	−	na	+	+	−	+	−	na	+	+
Delayed growth		+	+	+	na	+	−	+	−	−	+	na	+
Tonus disturbance		+	+	−	+	+	+	+	+	+	+	+	+
D. delay		+	+	−	na	+	+	+	+	+	na	+	+
**Encephalopathy**		+	+	+	na	+	+	−	+	−	+	+	+
**Liver failure**		+	+	+	+	−	−	+	−	+	na	na	−
Myopathy		−	−	−	−	−	−	−	+	−	na	na	−
Lactate ↑		+	+	+	+	+	+	+	+	+	na	na	+
OXPHOS ↓	Fib	IV, I, (III, V)	I, IV	I, IV, (III, V)	IV, (I, III, V)	I, (III−V)	I, III, IV	IV, (I)	I, IV	I, IV	IV	^*^	I, III, IV
	Mu	I, IV	−	IV, V, (I)	−	I, (IV)	III	−	IV	IV	IV	^*^	na
CNS	CC	+	+	+	−	−	+	−	−	na	na	na	+
Findings	WM	+	+	−	−	+	+	+	+	na	na	na	+
	BG	+	−	−	−	+	−	+	+	na	na	na	+
	BS	−	−	−	−	+	−	−	+	na	na	na	−
Age - onset		10d	7w	1d	*In utero*	3w	2d	2d	1d	2d	<1w	<1y	2m
Age–death		27d	5m	9d	<1d	16m	2y	8m	4y	20m	na	na	-

a *Coenen et al., 2004*.

b *Antonicka et al., 2006*.

c *Valente et al., 2007*.

d *Smits et al., 2011*.

e *Balasubramaniam et al., 2012*.

f *Galmiche et al., 2012*.

g *Calvo et al., 2012*.

h *this report*.

* *Described as “Combined OXPHOS deficiencies,” but respiratory chain enzyme analysis was only reported for liver cells (marked deficient complex I and IV activities)*.

*BG, Basal Ganglia; BS, Brainstem; CC, Corpus Callosum; CNS, Central Nervous System; d, days; D, Developmental; Fib, Fibroblasts; IU, Intrauterine; m, months old; Mu, Muscle; na, not applicable/not available; OXPHOS, Oxidative Phosphorylation; IUGR, intrauterine growth restriction; y, years old; w, weeks old; WM, White Matter; +, present; −, *absent**.

Similar to other described cases, our patient presented with early-onset failure to thrive, as well as encephalopathy and elevated blood lactate (Table 1). This latter biochemical finding was the only feature persistently evident in all previous patients with the exception of those reported by Calvo and colleagues who did not mention biochemical findings (Calvo et al., 2012). Smits and colleagues suggested that blood lactate levels correlated with disease severity (Smits et al., 2011), but this was not consistent in all the patients described. Our patient showed a maximum plasma lactate value of 4.87 mmol/L with initial responsive acidosis, whilst in other cases this ranged from 3.0 to 17 mmol/L with some children presenting with refractory fatal lactic acidemia.

Other common features were hypo- or hypertonia, developmental delay, seizures in three cases (Valente et al., 2007; Smits et al., 2011; Galmiche et al., 2012) and polyneuropathy (Galmiche et al., 2012). The early onset, rapidly progressive and fatal clinical course described in previously reported cases (Table 1) did not unfold in the patient we present, contrary to the hypothesis of Trivigno and Haerry (2011). In fact, having stabilized both clinically and biochemically, she now maintains a reasonable quality of life and shows no signs of imminent deterioration. However, despite her apparent clinical stability, there is some neuroradiological evidence that her condition continues to be slowly progressive (Figures 1E–F).

Microcephaly and minor dysmorphic traits, though not uncommon in early onset mitochondrial disease, are not uniformly described in this condition. Common neuroradiological features in the reported cases include corpus callosum thinning, leukodystrophy and basal ganglia involvement (Table 1), findings also identified in the patient we report. Cardiac function was preserved in all patients. The detailed clinical picture and outcome of the two unrelated patients mentioned by Calvo and colleagues were not thouroughly described in their report (Calvo et al., 2012), precluding a direct comparison at present.

Our patient is compound heterozygous for a novel frameshift mutation and a reported missense mutation, resulting in a severe reduction in steady-state levels of mtEFG1 protein. The c.1404delA, p.(Gly469Valfs^*^84) mutation has not previously been described, and introduces a premature termination codon into the *GFM1* transcript. Although not experimentally-confirmed, this mutation is defined as pathogenic and is highly likely to lead to nonsense-mediated mRNA decay. The c.2011C>T, p.(Arg671Cys) pathogenic mutation has previously been identified in the homozygous state in a patient presenting with a complex phenotype including severe encephalopathy and microcephaly, severely decreased complex IV activity in muscle and reduced activity of complexes I and IV in skin fibroblasts (Galmiche et al., 2012) and as a heterozygous recessive mutation by Calvo et al. (2012). Western blot analysis showed mtEFG1 protein reduced to <1% of control values in fibroblasts from the patient and there was also a marked decrease in full assembled complexes I, IV, and V compared to normal controls. The c.1404delA, p.(Gly469Valfs^*^84) and c.2011C>T, p.(Arg671Cys) mutations therefore seem highly likely to be causative of our patient's clinical presentation and multiple respiratory chain deficiency, indicating a disorder of generalized mitochondrial translation. A comparison with the cases reported in the literature, including two sibling pairs (Coenen et al., 2004; Antonicka et al., 2006), shows all patients have inherited *GFM1* mutations as recessive Mendelian traits, with clear loss of function alleles leading to mitochondrial translation defects with a combined deficiency of OXPHOS components (Table 2). For all described cases, OXPHOS anomalies were suspected histochemically by decreased COX activity in muscle and the combined deficiency of OXPHOS components and disorder of generalized mitochondrial translation was confirmed biochemically (Table 1). Complex II activity was consistently spared and no quantitative or qualitative mtDNA anomalies were found in the patients (Galmiche et al., 2012).

**Table 2:** **Summary of the mutations in GFM1 gene found in the reported patients**.

**Reported cases**	***GFM1* mutation**	**Type of mutation**
A^a^	Homozygous c.521A>G p.(Asn174Ser)	Missense
B^a^	Homozygous c.521A>G p.(Asn174Ser)	Missense
C^b^	c.961T>C p.(Ser321Pro) + c.1765-2_1765-1delAG p.(Gly589Profs^*^19)	Missense + Frameshift
D^b^	c. 961T>C p.(Ser321Pro) + c.1765-2_1765-1delAG p.(Gly589Profs^*^19)	Missense + Frameshift
E^c^	c.139C>T p.(Arg47^*^) + c.1487T>G p.(Met496Arg)	Nonsense + Missense
F^d^	Homozygous c.748C>T p.(Arg250Trp)	Missense
G^e^	c.539delG p.(Gly180Alafs^*^11) + c.688G>A p.(Gly230Ser)	Frameshift + Missense
H^f^	Homozygous c.2011C>T p.(Arg671Cys)	Missense
I^f^	Homozygous c.1193T>C p.(Leu398Pro)	Missense
J^g^	c.720delT p.(Glu241Asnfs^*^2) + c.2011C>T p.(Arg671Cys)	Frameshift + Missense
K^g^	c.720delT p.(Glu241Asnfs^*^2) + c.910A>G p.(Lys304Glu)	Frameshift + Missense
L^h^	c.1404delA p.(Gly469Valfs^*^84) + c.2011C>T p.(Arg671Cys)	Frameshift + Missense

a *Coenen et al., 2004*.

b *Antonicka et al., 2006*.

c *Valente et al., 2007*.

d *Smits et al., 2011*.

e *Balasubramaniam et al., 2012*.

f *Galmiche et al., 2012*.

g *Calvo et al., 2012*.

h *this report*.

*All mutations named according to reference NM_024996.5*.

Interestingly, the residual steady-state levels of mutated mtEFG1 protein have been found to be higher in heart and skeletal muscle than in liver and fibroblasts (Antonicka et al., 2006), which might diminish the susceptibility of these tissues, as has been observed in the published cases (Coenen et al., 2004; Antonicka et al., 2006; Smits et al., 2011; Trivigno and Haerry, 2011). Reports suggest that the severity of the OXPHOS defects due to *GFM1* mutations correlates with the residual level of the mutant protein in each tissue (Antonicka et al., 2006; Trivigno and Haerry, 2011; Balasubramaniam et al., 2012). The differences in residual protein levels are potentially due to diverse regulatory and compensatory responses of the mitochondrial translation system in different tissues that are currently not fully understood. Indeed, altered expression of other translation elongation factors (mtEFTu and mtEFTs) has been previously demonstrated in different tissues from a patient with *GFM1* mutations (Antonicka et al., 2006). The location of the specific mutation may affect particular functions and interactions of the protein, which may be tissue specific. For example, Galmiche and colleagues hypothesized that hepatic failure observed in some cases was particularly associated with mutations located in the central region of the mtEFG1 protein and those associated with encephalopathy were located in peripheral segments of the protein (Galmiche et al., 2012).

These concepts support previous clinical findings describing two main phenotypes, namely hepatic (Coenen et al., 2004; Antonicka et al., 2006; Balasubramaniam et al., 2012; Galmiche et al., 2012) and neurological (Valente et al., 2007; Smits et al., 2011; Galmiche et al., 2012) (Table 1). In keeping with this hypothesis, it is reasonable to suggest that the severe and exclusively neurological phenotype in our patient implies that the translation defect was most severe in the central nervous system.

The main reported causes of death have been related to respiratory complications (secondary to neurological dysfunction) and to multisystem failure secondary to severe hepatic dysfunction (Table 1). Despite the severity of the OXPHOS defects in mitochondria and the similarity of the results to the other patients, the progression of the disease appears to be more benign in our patient. At present, she is 5 years 6 months old and has a severe encephalopathy, but has had no additional episodes of acute clinical or metabolic decompensation since first coming to medical attention at the age of 7 months. Her general condition has stabilized, without regression, and she is able to be fed by mouth, maintaining normal growth. The specific functional and morphological consequences of the *GFM1* mutations identified may help explain the particular clinical course observed in this case, but there are still no definitive clinical, laboratory, radiological or genetic characteristics that could be helpful in predicting disease severity and prognosis in this condition.

Our understanding of the mitochondrial translation apparatus is increasing as more proteins are being identified in the pathways of mitochondrial gene expression, but these type of disorders remain without gene-specific treatment, much less a cure (Ribas et al., 2014; Soiferman et al., 2014; Szeto, 2014). Current investigations have evaluated the effect of small molecules - largely through the activation of the PGC1α pathway to stimulate mitochondrial biogenesis and consequently ATP synthesis - on improving mitochondrial function in fibroblasts of patients with combined respiratory complex disorders harboring known mutations in various nuclear-encoded components of the mitochondrial translation machinery. One such study included a patient with *GFM1* mutations and showed that the compound AICAR (5-aminoimidazole-4-carboxamide ribonucleotide) increased complex IV activity in *GFM1* cells, demonstrating therapeutic potential (Soiferman et al., 2014). This was one example of a specific benefit of one compound in a particular cell line, but no universally beneficial compound was found, highlighting the importance of personalized medicine in mitochondrial disease (Soiferman et al., 2014).

## Concluding remarks

Mutations in *GFM1* gene are consistently associated with a combined respiratory chain deficiency and persistent elevation of blood lactate. Clinical findings are heterogeneous, but neurological and hepatic involvement are both prominent features. Although the previously reported cases consisted of early onset, rapidly progressive and ultimately fatal diseases, the patient described here seems to show a more benign phenotype, though the pathophysiology and prognosis remain unclear. To our knowledge, this is the first patient with recessive *GFM1* mutations who has survived beyond early childhood.

### Conflict of interest statement

The Reviewer Veronika Boczonadi declares that, despite being affiliated to the same institution as the authors Kyle Thompson, Steven A. Hardy, Langping He, and Robert McFarland, the review process was handled objectively and no conflict of interest. The authors declare that the research was conducted in the absence of any commercial or financial relationships that could be construed as a potential conflict of interest.
