# Corrigendum: Long-term survival in a child with severe encephalopathy, multiple respiratory chain deficiency and *GFM1* mutations

**DOI:** 10.3389/fgene.2015.00254

**Published:** 2015-07-28

**Authors:** Sara Brito, Kyle Thompson, Jaume Campistol, Jaime Colomer, Steven A. Hardy, Langping He, Ana Fernández-Marmiesse, Lourdes Palacios, Cristina Jou, Cecilia Jiménez-Mallebrera, Judith Armstrong, Raquel Montero, Rafael Artuch, Christin Tischner, Tina Wenz, Robert McFarland, Robert W. Taylor

**Affiliations:** ^1^Serviço de Pediatria, Centro Hospitalar de Leiria, Hospital de Santo AndréLeiria, Portugal; ^2^Neuromuscular Unit, Neuropaediatrics Department, Hospital Sant Joan de DéuBarcelona, Spain; ^3^Wellcome Trust Centre for Mitochondrial Research, Institute of Neuroscience, Newcastle UniversityNewcastle upon Tyne, UK; ^4^Centro de Investigación Biomédica en Red de Enfermedades Raras, Instituto de Salud Carlos IIIBarcelona, Spain; ^5^Diagnosis and Treatment Unit for Inborn Errors of Metabolism, Hospital Clínico Universitario de Santiago de CompostelaLa Coruña, Spain; ^6^Progenika Biopharma a Grifols CompanyDerio, Spain; ^7^Pathology Department, Hospital Sant Joan de DéuEsplugues Barcelona, Spain; ^8^Biochemical, Genetics and Rett Unit, Laboratory Department, Hospital Sant Joan de DéuEsplugues Barcelona, Spain; ^9^Biochemical Department, Hospital Sant Joan de DéuEsplugues Barcelona, Spain; ^10^Cluster of Excellence, Cellular Stress Responses in Aging-Associated Diseases, Institute for Genetics, University of CologneCologne, Germany; ^11^German Network for Mitochondrial DisordersMunich, Germany

**Keywords:** *GFM1*, mtEFG1, mitochondrial disorders, brain MRI, encephalopathy

In our article entitled, “**Long-term survival in a child with severe encephalopathy, multiple respiratory chain deficiency and *GFM1* mutations**,” published in *Frontiers in Genetics* on 23rd March 2015 we inadvertently omitted information that the attending paediatrician believes may be relevant to the case. In the interests of accuracy and completeness we would like to add that the patient we describe had been receiving treatment with Ubiquinone (20 mg/day), Riboflavin (100 mg/day), Carnitine (500 mg/day), N-acetylcysteine (100 mg/day), and Folinic acid (15 mg/day) for 6 months prior to inclusion in the report. While there was no obvious improvement in her condition, equally she has not exhibited any further clinical deterioration since treatment began. Radiological progression was, however, evident on a cranial MRI performed 5 months after treatment was initiated. Given the relatively short duration of treatment (approximately 6 months at the time of writing the report) and the neuroradiological progression evident on the repeat cranial MRI, we consider it unlikely that this vitamin and antioxidant treatment has made a substantial contribution to the patient's longer survival.

## Conflict of interest statement

The authors declare that the research was conducted in the absence of any commercial or financial relationships that could be construed as a potential conflict of interest.

